# Van Wyk-Grumbach Syndrome with bilateral inguinal hernia: A case report

**DOI:** 10.1016/j.ijscr.2025.110975

**Published:** 2025-01-28

**Authors:** Dhiran Sivasubramanian, Virushnee Senthilkumar, Smrti Aravind, Aswin Ram Rajasekar, Sharan Prasaanth, Sathwik Sanil

**Affiliations:** a Department of Critical Care Medicine, Christian Medical College, Vellore, India; b Coimbatore Medical College and Hospital, Coimbatore, India; c Institute of Oncology, Sri Ramakrishna Hospital, Coimbatore, India

**Keywords:** Van Wyk-Grumbach Syndrome, Juvenile hypothyroidism, Ovarian cysts, Inguinal hernia, Case report

## Abstract

**Introduction:**

Van Wyk-Grumbach Syndrome (VWGS) is a rare pediatric endocrinological disorder characterized by hypothyroidism, delayed bone age, enlarged multicystic ovaries, and precocious puberty. It results from prolonged hypothyroidism, affecting the hypothalamic-pituitary-gonadal axis. This report describes a 7-year-old girl presenting with vaginal bleeding and abdominal pain, leading to a diagnosis of VWGS with bilateral inguinal hernia and requiring surgical intervention.

**Case presentation:**

A 7-year-old girl presented with a single episode of vaginal bleeding, abdominal pain, and growth retardation. Clinical examination revealed delayed growth parameters, Tanner stage II breast development, and bilateral inguinal hernias. Abdominal examination identified a cystic mass in the right iliac fossa. Laboratory tests showed hypothyroidism. Imaging revealed a multiloculated right ovarian cyst. The patient underwent exploratory laparotomy with right salpingo-oophorectomy, marsupialization of the left ovarian cyst, and bilateral hernia repair. Histopathology confirmed ovarian hemorrhagic infarction. Postoperatively, she was started on levothyroxine therapy, leading to symptom resolution, height improvement, and cyst regression during follow-up.

**Clinical discussion:**

VWGS manifests due to thyroid dysfunction-induced gonadal stimulation, causing ovarian enlargement and precocious puberty. Prompt initiation of levothyroxine can prevent complications and avoid surgical intervention. The bilateral hernias in this case represent a unique presentation, potentially linked to hypothyroidism-induced muscle weakness.

**Conclusion:**

This case underscores the necessity of routine thyroid evaluation in pediatric patients with ovarian cysts and precocious puberty. Early diagnosis and levothyroxine therapy can resolve symptoms and prevent invasive interventions, emphasizing the critical role of endocrinological assessment.

## Highlights

• First reported case of Van Wyk-Grumbach Syndrome (VWGS) with bilateral inguinal hernias
• Hypothyroidism-induced ovarian hyperstimulation causing precocious puberty
• Surgical intervention included salpingo-oophorectomy and hernia repair.
• Need for early recognition and prompt initiation of levothyroxine therapy to avoid unnecessary gonadal surgery
• Importance of surgery for acute complications

## 1 Introduction

Van Wyk-Grumbach Syndrome (VWGS) is a rare endocrinological condition characterized by hypothyroidism, delayed bone age, enlarged multicystic ovaries, and precocious puberty [1]. Ovarian cysts in the pediatric population must be carefully evaluated as they often arise secondary to some endocrine disorder [1]. The pathophysiology is not completely understood but is believed to be related to the effects of long-standing hypothyroidism on the hypothalamic-pituitary-gonadal axis [2]. This report discusses a case of VWGS in a 7-year-old girl presenting with vaginal bleeding and abdominal pain, who was subsequently diagnosed with Van Wyk-Grumbach Syndrome and underwent surgical intervention for alleviation of her symptoms.

## 2 Methods

This work was completed in line with the SCARE criteria [14].

## 3 Case presentation

A 7-year-old female Indian child presented to the emergency department with chief complaints of vaginal bleeding and abdominal pain lasting one day. Her parents reported that she was not gaining height adequately. The parents were of normal height and weight. There was no history of local trauma, bleeding manifestations, difficulty in micturition, galactorrhea, headache, or visual disturbance. There was no family history of pubertal precocity or thyroid disease.

On physical examination, she was noted to be alert and oriented. Her height was <84 cm (less than 3rd percentile for her age) with an upper segment to lower segment ratio 1.47 (normal 1.1). Her weight was 18 kg (below the 25th percentile for her age). Breast development was consistent with Tanner stage II. There was no evidence of goitre, or axillary and pubic hair development. Vitals at presentation were a heart rate of 64 beats/min, blood pressure was 110/60 mmHg, respiratory rate at rest was 16 /minute, and oxygen saturation on room air was 100 %. Audiological and visual assessment results were normal.

Abdominal examination revealed a firm, mobile mass measuring approximately 10 × 7 × 5 cm, palpable in the right iliac fossa and extending into the supraumbilical region. The mass had well-defined borders and exhibited a smooth surface. Additionally, bilateral inguinal hernias with positive cough impulses were noted. Digital rectal examination (DRE) revealed hard stools, and external genital examination showed well developed labia, clitoris and anal orifice, excluding local causes of bleeding, confirming vaginal bleeding.

**Fig. 1:**
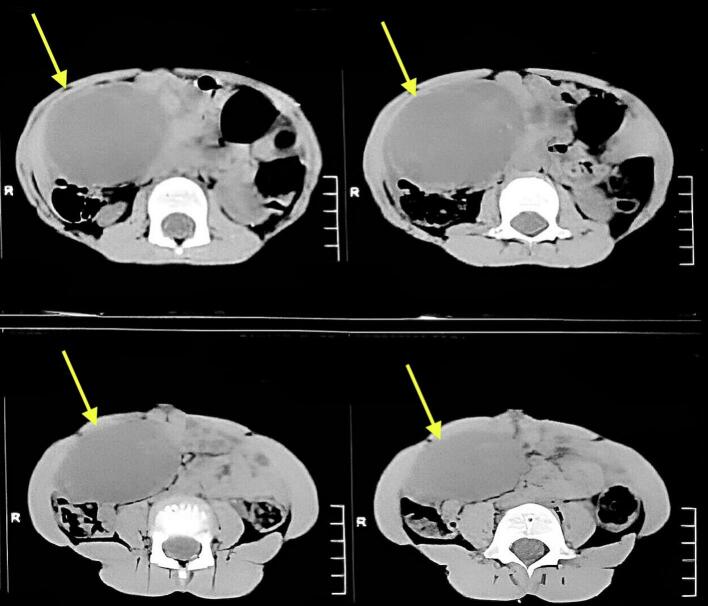
Contrast enhanced computed tomography (CECT) of the abdomen and pelvis showing multiloculated cystic lesion measuring 10 × 7.2 × 5 cm with internal septation possibly arising from the right ovary.

Laboratory investigations revealed an elevated TSH of >100 mIU/mL, with a free T4 level of 0.4 ng/dL (0.7–1.8 ng/dL). Ultrasonography (USG) of the abdomen and pelvis identified a well-defined cystic lesion measuring 8.7 × 4 × 8.3 cm in the abdominopelvic region. Subsequent contrast-enhanced computed tomography (CECT) of the abdomen and pelvis revealed a multiloculated cystic lesion with internal septations arising from the right ovary, indicative of a right ovarian cystic mass (Fig. 1).

**Fig. 2:**
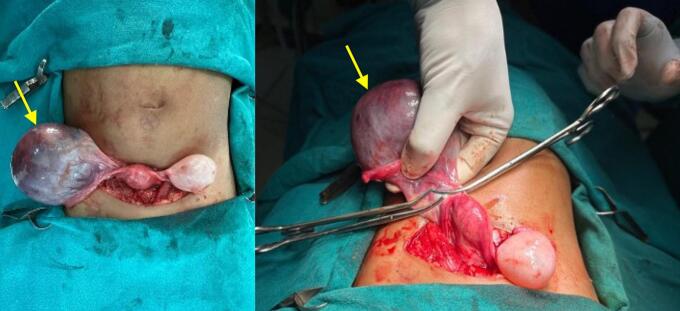
Intraoperative findings showing: an ischemic right ovarian mass with a cystic component (yellow arrow) and a left ovarian cyst.

**Fig. 3:**
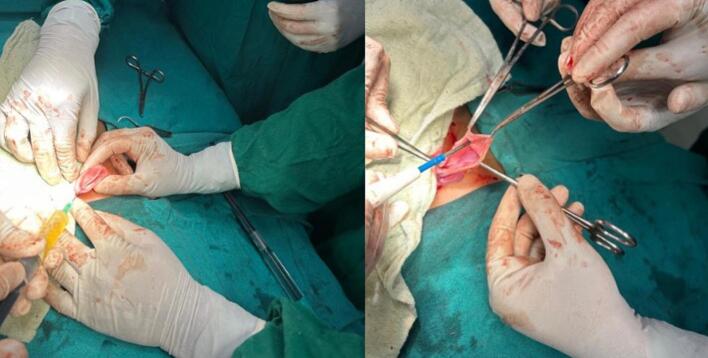
Drainage and marsupialization of the left ovarian cyst wall.

Given the findings of abdominal pain, a significant ovarian mass, and hypothyroidism, surgical intervention was planned. The patient was started on levothyroxine daily for hypothyroidism and underwent an exploratory laparotomy. Intraoperative findings included: a right ovarian mass with a cystic component which appeared severely ischemic, a left ovarian cyst and bilateral inguinal hernias (Fig. 2). Right salpingo-oophorectomy with marsupialization of the left ovarian cyst wall, and bilateral deep ring narrowing was performed (Fig. 3).

Histopathological exam of the right ovarian mass revealed hemorrhagic infarction of the ovary. The patient was discharged in stable condition and continued on levothyroxine therapy, and advised to follow up regularly. On follow-up, she exhibited marked improvement in her symptoms. Her height increased by 2 cm over the first 3 months and another 4 cm over the subsequent 3 months. The ovarian cysts did not recur and breast size regressed, and she did not experience further menstruation.

## 4 Discussion

Van Wyk-Grumbach Syndrome (VWGS) was described by Van Wyk and Grumbach in 3 girls with Juvenile hypothyroidism in the 1960s in which the cardinal feature is sexual development beyond that consistent with the bone age and other indices of maturity [3]. VWGS predominantly occurs in female children accounting for about 92.7 % of the cases with a median diagnosis age of 8.5 years [4] but also few cases were reported in male children presenting with testicular enlargement and minimal penile enlargement [5]. It is postulated that the association of hypergonadism and precocious puberty was due to the non-specific nature of the pituitary feedback mechanism leading to over secretion of TSH and gonadotropins [3]. These high levels of FSH and LH along with the action of increased levels of TSH, which can act on the FSH receptors result in ovarian hyperstimulation, leading to precocious puberty, enlargement of the ovaries and the uterus resulting in menorrhagia. The main clinical features of VWGS include delayed bone age, enlarged multicystic ovaries, and precocious puberty. It often presents as early breast development and menstruation, although some patients may present without secondary sexual characteristics as seen in our patient. Due to a lack of stimulation for adrenarche, pubic and axillary hair development does not occur in these patients. High levels of TRH stimulate high prolactin levels leading to galactorrhoea and the lack of negative thyroid feedback leads to thyrotrophic stimulation sometimes causing pituitary hyperplasia [6]. The laboratory findings for these patients can be characterized by high levels of TSH, low free thyroxine, and abnormal levels of FSH and LH.

Bilateral hernia has not yet been reported in the literature involving Van Wyk Grumbach syndrome; this specific clinical presentation can be due to the increased intra-abdominal pressure that has developed as the result of the indolent growing abdominal mass. A study by Salomonowitz et al. in 1983 found that among 25 patients with symptomatic hernias over a 10-year period, 92 % of them were associated with intraabdominal masses that were malignant, with 88 % located in the pelvis further more establishing this concept [7]. Also, hypothyroidism can cause muscle weakness and hypotonia, which may affect the integrity of the abdominal wall since thyroid hormone is associated with the muscle mass maintenance and protein turnover [8]. These factors such as increased intra-abdominal mass, constipation due to hypothyroidism, and muscle weakness may have contributed to the development of bilateral hernias in our patient. A case of umbilical hernia with VWGS was reported by Dutta et al. in 2013 [9]. Our patient was treated for bilateral inguinal hernia surgically when treating for the acute surgical abdomen due to infarcted right ovarian mass.

The early detection of this condition is necessary to prevent the unfortunate event of removal of the gonadal tissue. Regular pediatric evaluations could reveal growth restriction and early signs of puberty. The presence of features of hypothyroidism, delayed bone growth, and precocious puberty in a child should raise suspicion of VWGS. Further evaluation for pelvic mass through imaging should be considered, to prevent such catastrophical outcomes. Surgical treatment such as cystic puncture and ovarian resection could be avoided with prompt initiation of treatment with levothyroxine, a case series of 10 children with multicystic ovaries, which regressed after administering thyroxine in 3 to 6 months [10]. There were cases that presented with acute surgical abdomen and had to be treated with salpingo-oophorectomy, in a case series done in India by Boddu et al. [11], among 27 girls, 7 presented with the acute surgical abdomen in which 2 required immediate surgery due to ovarian torsion. Some cases of VWGS have elevated levels of CA-125, alpha-fetoprotein, and lactate dehydrogenase which all normalized after prompt treatment [12,13]. Hence, these masses could also mimic ovarian malignancies prompting the consideration of surgical treatment.

The severity of our patient's abdominal pain led to the suspicion of ischemia which was later histopathologically confirmed by hemorrhagic infarction in the right ovary. The patient's severe symptoms, including pain, a large ovarian mass, and bilateral inguinal hernias, necessitated surgical management.

## 5 Conclusion

This case highlights the importance of considering endocrine disorders in pediatric patients with ovarian cysts and precocious puberty. While surgical intervention may be necessary for acute complications, timely detection of hypothyroidism and initiation of levothyroxine therapy often resolves the ovarian cysts and mitigates complications, underscoring the importance of routine thyroid, growth and abdominopelvic evaluations in similar presentations.

## Consent

Written informed consent was obtained from the patient's guardian for publication of this case report and accompanying images. A copy of the written consent is available for review on request.

## Ethical approval

All the data of this study was taken from the medical records of the patient. This report does not contain any personal information that could lead to the identification of the patient. Therefore, it is exempted from ethical approval.

## Guarantor

Dhiran Sivasubramanian

Virushnee Senthilkumar

Smrti Aravind

Aswin Ram Rajasekar

Sharan Prasaanth

Sathwik Sanil

## Funding

No funding was provided for the completion of this manuscript.

## Author contribution

Dhiran Sivasubramanian - Conceptualization, Writing - Original Draft, Writing - Review & Editing, Project Administration.

Virushnee Senthilkumar - Writing - Original Draft, Writing - Review & Editing.

Smrti Aravind - Writing - Original Draft, Writing - Review & Editing.

Aswin Ram Rajasekar- Writing - Original Draft, Writing - Review & Editing.

Sharan Prasaanth- Writing - Original Draft, Writing - Review & Editing.

Sathwik Sanil - Writing - Original Draft, Writing - Review & Editing.

## Conflict of interest statement

We, the authors of this article, declare that we have no known competing financial interests or personal relationships that could have appeared to influence the work reported in this paper.
